# Reconstruction of Immune Microenvironment and Signaling Pathways in Endometrioid Endometrial Adenocarcinoma During Formation of Lymphovascular Space Involvement and Lymph Node Metastasis

**DOI:** 10.3389/fonc.2020.595082

**Published:** 2020-12-09

**Authors:** Yuan Cheng, Xiaobo Zhang, Zhiqi Wang, Jianliu Wang

**Affiliations:** ^1^ Department of Obstetrics and Gynecology, Peking University People’s Hospital, Beijing, China; ^2^ Department of Pathology, Peking University People’s Hospital, Beijing, China

**Keywords:** lymphovascular space involvement, lymph node metastasis, immune surveillance, neutrophils, macrophages, mast cells, endometrioid endometrial adenocarcinoma

## Abstract

**Background:**

The amplification or mutation of oncogenes and escape from immune surveillance systems promote tumor metastasis. However, subtle changes in the immune microenvironment and signaling pathways are poorly understood during the formation of lymphovascular space involvement (LVSI) and lymph node (LN) metastasis of endometrioid endometrial adenocarcinoma (EEA).

**Patients and methods:**

We detected tumor immunology-related signaling pathways and immunocyte subtypes according to the mRNA levels of 750 oncogenes and genes relating to the tumor microenvironment and immune response using the Nanostring PanCancer IO 360 Panel in 24 paraffin-embedded tissues of EEAs and benign gynecological diseases. Internal reference genes were used for data normalization.

**Results:**

Angiogenesis and immune cell adhesion signaling pathways were activated during LVSI formation of EEA progression. However, during the development of LVSI to LN metastasis, immune system signaling pathways were significantly inhibited, including antigen presentation, cytotoxicity, lymphoid compartment, interferon signaling, and costimulatory signaling pathways. Immune-related genes (CD69, HLA-DOA, ATF3, GBP1, AP2, DTX3L, EGR1, GBP4, TAP1, EIF2AK2, MX1, ISG15, STAT1, and HLA-DRA) were significantly downregulated in EEA with LN metastasis compared to those in EEA with LVSI. Instead, hypoxia, metabolic stress, epigenetic regulation, matrix remodeling, and metastasis signaling pathways were continuously activated in LN metastasis. We also found that neutrophils, macrophages, and mast cells might be involved in LVSI formation and LN metastasis in EEA.

**Conclusions:**

EEA with metastatic LNs showed significant immunosuppressive effects. Some oncogenes, matrix remodeling- and hypoxia-related genes, and neutrophil signatures showed higher expression, suggesting their potential as therapeutic targets and offering new immunotherapy strategies in EEA during LN metastasis.

## Introduction

Endometrial cancer (EC) is ranked 4th and 6th in morbidity and mortality, respectively, among cancers affecting women in the United States in 2019 (1). Statistically, the mortality rate of EC slowly increased from 2012 to 2016 in China (2). The 5-year survival rates of patients with endometrial carcinoma at FIGO stages III and IV were only 57–66% and 20–26%, respectively (3, 4). Endometrial carcinoma with lymphovascular space involvement (LVSI), myometrial invasion, lymph node (LN) metastasis, and high-grade cancer were associated with significantly higher recurrence rates (5). Accumulated studies have shown that LN metastasis is a strong independent prognostic factor for endometrial carcinoma recurrence (6, 7). Recent studies revealed that LVSI is an independent prognostic factor for lymph node metastasis and non-locoregional recurrence in early-stage endometrial carcinoma (8, 9). However, little is known about the regulation of molecular mechanisms and the tumor microenvironment for LVSI and LN metastasis in endometrial carcinoma.

The immune surveillance system and cancer cells fight a seesaw-like battle from occurrence to the early and advanced stages. In the early stage, the immune system produces an anti-inflammatory microenvironment to fight against cancer cells, whereas in the late stage, tumor cells escape immune surveillance, resulting in distant metastasis and recurrence (10). Antomarchi etal. (11) found that grade 1 ECs showed a strong anti-tumor immune microenvironment; however, the high-grade ECs presented immunotolerance and immunosuppression. Pakish etal. (12) showed increased infiltration of immune cells, including granzyme B+ cells, activated cytotoxic T lymphocytes, and PD-L1+ cells, in endometrial carcinomas with high microsatellite instability (MSI-H) compared to those with microsatellite stability (MSS). These studies suggested that low-grade tumors and MSI-positive endometrial carcinoma might be more sensitive to immunotherapy. However, the regulation of the immune microenvironment and signaling pathways remain poorly understood in relation to LVSI formation and LN metastasis of endometrial carcinoma.

Immune system surveillance has been shown to play a role in type I endometrioid endometrial adenocarcinoma (EEA), while it is inert in type II serous carcinoma (13, 14). This study focused on the construction of the spectrum of tumor immune microenvironments of EEA during LVSI formation and LN metastasis. It was determined that immune system activation was present in EEAs with LVSI formation. However, severe immunosuppression and tolerance were observed in ECs with LN metastasis. Hypoxia, metabolic stress, epigenetic regulation, matrix remodeling, and metastasis signaling pathway-related oncogenes and neutrophil signatures showed higher expression, suggesting their potential as therapeutic targets and offering new immunotherapy strategies for LN metastasis in EEA.

## Methods

### Patients and Specimens

Paraffin sections from 24 patients with EEA and benign gynecological disease were obtained from the pathology department of Peking University People’s Hospital. LVSI and LN metastasis were important pathological progress indicators of EEA. Cases were separated into 4 groups of 6 cases: Normal control, LVSI-LN-, LVSI+LN-, and LVSI+LN+. The clinicopathological data of 18 EEAs are listed in **Table 1**. LVSI and LN metastasis of endometrial carcinoma were re-identified by a senior pathologist. The study was approved by the ethics committee of Peking University People’s Hospital (2019PHB031-01).

**Table 1:** The clinicopathological characteristics of 18 endometrial cancer patients.

Variables	n=18 (No. of patients) %
Age (median)	54 years (49–63 years)
FIGO stage	
I	12 (66.7)
III	6 (33.3)
Histological grade	
Grade 1	4 (22.2)
Grade 2	9 (50.0)
Grade 3	5 (27.8)
Tumor diameter	
<2	4 (22.2)
≥2	14 (77.8)
LVSI	
No	6 (33.3)
Yes	12 (66.7)
LN metastasis	
No	12 (66.7)
Yes	6 (33.3)

Data are expressed as n (%). Abbreviations: FIGO, International Federation of Gynecology and Obstetrics; LVSI, lymphovascular space involvement; LN, lymph node.

### Analysis of mRNA Expression

There or four formalin-fixed, paraffin-embedded curls (10 µm; effective tissue sample area > 1.5 × 1.5 cm) were prepared for RNA extraction using the High Pure RNA Paraffin Kit (Roche Applied Science, Penzberg, Germany). RNAs were at least 300 nt in length, and ≥50% RNA content was obtained. At least 300 ng total RNA was obtained per sample. RNA quality was detected by Nanodrop (A260/A280: 1.7–2.3; A260/A230: 1.8–2.3). The tumor-related signaling pathways and tumor-infiltrating lymphocyte (TIL) counts were examined according to the mRNA levels of 750 genes relating to the tumor microenvironment and immune response as well as internal reference genes for data normalization using the Nanostring PanCancer IO 360 Panel (Agilent 2100 Bioanalyzer and an Agilent RNA 6000 Nano Kit, Nanostring Technologies, Inc., Seattle, WA, USA); (https://www.nanostring.com/products/gene-expression-panels/gene-expression-panels-overview/360-series-panel-collection/pancancer-io360-gene-expression-panel). These 770 genes were classified into13 annotations and selected reference genes as follows: release of cancer cell antigens (74), cancer antigen presentation (101), T-cell priming and activation (150), immune cell localization to tumors (292), stromal factors (102), recognition of cancer cells by T-cells (105), killing of cancer cells (179), myeloid cell activity (260), natural killer (NK) cell activity (28), cell cycle and proliferation (54), tumor-intrinsic factors (155), immunometabolism (101), common signaling pathways (162), and internal reference genes (20).

Genes were classified as 14 immune cell type metagenes (B-cells, CD45, CD8 T cells, cytotoxic cells, dendritic cells [DCs], exhausted CD8, macrophages, mast cells, neutrophils, NK CD56dim cells, NK cells, T cells, Th1 cells, and Tregs). Besides, 39 signaling pathways were analyzed in 13 annotations as follows: release of cancer cell antigens (microsatellite instability/MSI, double strand break repair, chromatin modification/epigenetics, DNA damage repair), cancer antigen presentation (MHC class-I/II genes, non-MHC antigen presentation, antigen processing machinery, proteasome and immunoproteosome, cross-presenting DC genes), T-cell priming and activation (costimulatory molecules), immune cell localization to tumors (chemokines, integrins, selectins, immune cell populations in tumors), stromal factors (extracellular matrix remodeling, collagens, angiogenesis, metastasis), recognition of cancer cells by T-cells (immune checkpoints), killing of cancer cells (interferon signaling, JAK-STAT1/2 pathway, cytolytic activity, phagocytosis), myeloid cell activity (inflammation, Fc-gamma receptor signaling), NK cell activity, cell cycle and proliferation, tumor-intrinsic factors (apoptosis, autophagy, nutrient depletion, metastasis), immunometabolism (oxygen sensing, nutrient regulation), and common signaling pathways (Wnt, Hedgehog, TGF-β, NF-κB, Notch, PI3K-Akt, RAS, MAPK).

### Bioinformatics and Statistical Analysis

The raw gene expression values were normalized to those of housekeeping genes using the NanoString nSolver 4.0 software (nanoString, Seattle, WA) and log2 transformation. Differentially expressed genes, along with false discovery rate (FDR) corrected p-values was screened out for signaling pathway analysis according to Kyoto Encyclopedia of Genes and Genomes (KEGG) pathways. Signaling pathway scores were clustered by unsupervised hierarchical clustering, and the 1-euclidean distance was used as the similarity measure. The P-values were adjusted using the Benjamini-Yekutieli (BY) false discovery rate and the Bonferroni correction. The false discovery rate was limited to ≤5% using P-values. Furthermore, the absolute and relative abundance of an immune cell subtype was estimated by simply taking the average log2 expression of the characteristic genes. Along with nanoString n-Solver software, one-way ANONA were used for statistical analysis. P-value for significance was set at p<0.05. Scatter plots charts were obtained using Graphpad Prism 8.

### Immunohistochemical Staining

Three-μm-thick slices stained with H&E or immunohistochemical (IHC) were obtained from formalin-fixed paraffin-embedded (FFPE) EEAs tissues, which were the same wax blocks as the previous nanostring analysis. IHC staining of FFPE slides was performed using monoclonal mAbs against BIRC5(ab76424), CD68(ab213363) and CD163(ab213612) (Abcam, Cambridge, UK), with 1:500 working dilution. Paraffin sections are first dewaxed in steps from xylene to different concentrations of alcohol, and finally placed in tap water for washing. The slices were treated with heated citric acid repair fluid for antigen repair. After incubation with 3% H2O2 for 10 min, endogenous peroxidase was removed. Goat serum was used for blocking for 30 min. The first antibody was incubated overnight at 4 degrees. The next day, the second antibody labeled with horseradish peroxidase was incubated for 30 min. In the middle of each step, 1 x PBS should be used for cleaning at 5 min * 3 times. DAB was used for IHC staining observed under the microscope.

Scoring for BIRC5, CD68, and CD163 was evaluated by percentage of cells stained in tumor and stromal tissue compartments by a pathologist.

## Results

### Heatmap of Signaling Pathway and Differentially Expressed Genes (DEGs) Between EEAs and Benign Gynecological Lesions

**Figure 1:**
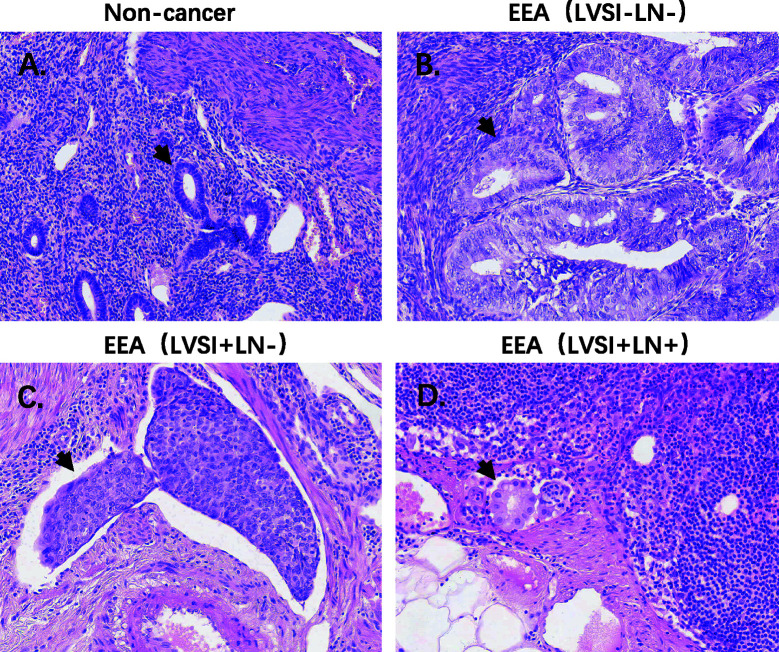
HE staining of endometrioid endometrial adenocarcinomas (EEAs) and benign gynecological lesions in 4 groups of 6 cases. LVSI, lymphovascular space involvement; LN, lymph node. **(A)** Non-cancer group. The black arrow refers to the normal gland; **(B)** LVSI-LN-group, the black arrow refers to the gland of EEA; **(C)** LVSI+LN-group, the black arrow refers to the LVSI. **(D)** LVSI+LN+ group, the black arrow refers to EEAs in LN.

**Figure 2:**
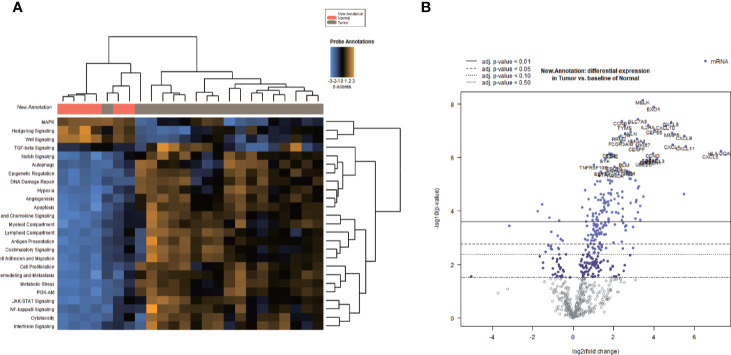
Heatmap of signaling pathway and differentially expressed genes (DEGs) between endometrioid endometrial adenocarcinomas (EEAs) and benign gynecological lesions. **(A)** The heatmap of all signaling pathways in EEAs compared to those of the benign gynecological lesions Red, gray bars represent normal and tumor, respectively. The transition between blue, black, and yellow represents the level scores of signaling pathways ranging from −3 to 3. Vertical lines or horizontally focused lines represent unsupervised clustering. In total, 25 signaling pathways are marked on the left side of the heat map. **(B)** DEGs between EEAs and benign gynecological lesions. Blue and purple dots represent DEGs, and a dot represents a gene. The left side of 0 represents the down regulated mRNA, and the right side of 0 represents the up-regulated mRNA on the X-axis. The log2 expression value of the gene corresponds to the Y-axis. Four different virtual and real lines represent different stages of P value, from P < 0.5 to P < 0.01.

HE staining from 24 patients with EEA and benign gynecological disease was showed in **Figure 1**. Cases were separated into 4 groups of 6 cases: Normal control (Non-cancer), LVSI-LN-, LVSI+LN-, and LVSI+LN+. The clinicopathological data of 18 EEAs are listed in **Table 1**. First of all, we analyzed the signal pathways and differentially expressed genes between 6 cases of normal control group and 18 cases of EEAs. The data showed that MAPK, Hedgehog signaling, Wnt signaling were significantly suppressed in EEAs. The other 22 signaling pathways, including cell proliferation, DNA damage repair and so on, are greatly activated in EEAs (**Figure 2A**). Differentially expressed genes (DEGs) between EEAs and normal control were showed in **Figure 2B**. Top 20 DEGs including MELK, EXO1, SLC7A5, CCNB1, CXCL8, IL2RA, TYMS, CXCL10, CEP55, ANLN, MMP9, IL7R, CXCL9, RRM2, HMGA1, FCGR3A/B, MKI67, CXCL1, CXCL11, CENPF were showed in **Table 2**.

**Table 2:** Top 20 differentially expressed mRNA in endometrioid endometrial adenocarcinomas (EEAs) compared to benign gynecological lesions.

Gene name	Log2 fold change	P-value	Gene.sets
MELK	3.45	7.27E-09	Cell Proliferation
EXO1	3.97	1.27E-08	Cell Proliferation, DNA Damage Repair
SLC7A5	3.2	3.73E-08	Metabolic Stress
CCNB1	2.4	4.46E-08	Cell Proliferation
CXCL8	4.85	4.77E-08	Cytokine and Chemokine Signaling, Metabolic Stress
IL2RA	3.72	6.18E-08	Costimulatory Signaling, Cytokine and Chemokine Signaling, JAK-STAT Signaling, PI3K-Akt
TYMS	2.54	6.36E-08	Cell Proliferation
CXCL10	4.57	6.37E-08	Cytokine and Chemokine Signaling, Lymphoid Compartment
CEP55	4.03	9.46E-08	Cell Proliferation
ANLN	2.85	1.05E-07	Cell Proliferation
MMP9	4.9	1.19E-07	Angiogenesis, Immune Cell Adhesion and Migration, Matrix Remodeling and Metastasis
IL7R	2.57	1.26E-07	Cytokine and Chemokine Signaling, JAK-STAT Signaling, PI3K-Akt
CXCL9	5.5	1.53E-07	Cytokine and Chemokine Signaling, Lymphoid Compartment
RRM2	2.29	1.7E-07	Cell Proliferation
HMGA1	3.16	1.99E-07	Epigenetic Regulation, Metabolic Stress
FCGR3A/B	2.39	2.62E-07	
MKI67	3.47	2.81E-07	Cell Proliferation
CXCL1	4.91	3.35E-07	Antigen Presentation, Cytokine and Chemokine Signaling, Myeloid Compartment
CXCL11	5.56	3.77E-07	Cytokine and Chemokine Signaling, Lymphoid Compartment
CENPF	3.15	4.11E-07	Cell Proliferation

### Signaling Pathway Characteristics of EEA During LVSI Formation and LN Metastasis

**Figure 3:**
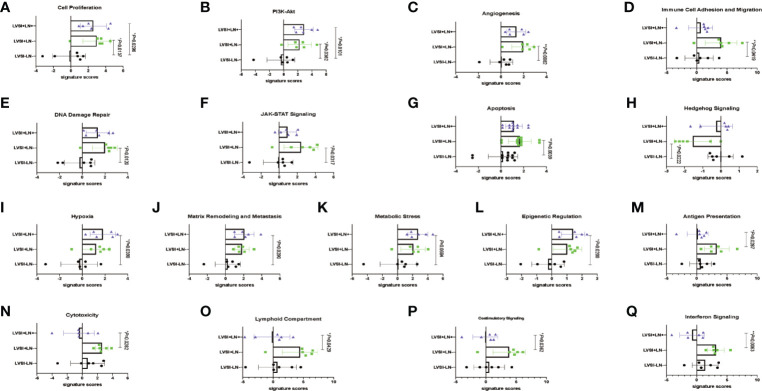
Changes of signaling pathways in lymphovascular space involvement (LVSI) formation and lymph node (LN) metastasis in endometrioid endometrial adenocarcinomas (EEAs) progression. Points represent the number of cases. Each group has 6 cases. Black, green, and purple represent LVSI-LN-, LVSI+LN-, and LVSI+LN+, respectively. **(A)** Cell proliferation **(B)** PI3K-Akt. **(C)** Angiogenesis. **(D)** Immune cell adhesion and migration. **(E)** DNA damage repair. **(F)** JAK-STAT signaling. **(G)** Apoptosis. **(H)** Hedgehog signaling. **(I)** Hypoxia. **(J)** Matrix remodeling and metastasis. **(K)** Metabolic stress. **(L)** Epigenetic regulation. **(M)** Antigen presentation. **(N)** Cytotoxicity. **(O)** Costimulatory signaling. **(P)** Interferon signaling. *P < 0.05, **P < 0.01.

Signaling pathways involved in LVSI formation and LN metastasis compared to LVSI-LN- group were shown in **Figure 3**. We found significantly different distributions of signaling pathways among the three groups (LVSI-LN-, LVSI+LN-, and LVSI+LN+) in EEA progression. Persistent activation of cell proliferation and PI3K-AKT signaling pathways was observed in the LVSI+LN- group LVSI+LN+ group compared to the LVSI-LN- group without metastasis in EEA progression (**Figures 3A, B)**. Hedgehog signaling pathways were downregulated whereas the angiogenesis, immune cell adhesion, apoptosis, DNA damage repair, and JAK-STAT signaling pathways were upregulated in EEA with LVSI compared to those in the LVSI-LN- group without metastasis (**Figures 3C–H**). Hypoxia, metabolic stress, epigenetic regulation, matrix remodeling, and metastasis signaling pathways were upregulated in EEA with LN metastasis compared to the LVSI-LN- group without metastasis (**Figures 3I–M)**. However, antigen presentation, cytotoxicity, lymphoid compartment, interferon, and costimulatory signaling pathways were all downregulated in EEA with LN metastasis compared to EEA with LVSI (**Figures 3N–Q)**. Cytokine and chemokine, myeloid compartment, autophagy, TGF-β, MAPK, NF-κB, Notch, and Wnt signaling pathways showed no significant changes in the absence of LVSI, LVSI formation, and LN metastasis during EEA progression (**Supplementary Figure 1**).

### Regulation of Critical mRNA Expression in EEA During LVSI Formation and LN Metastasis

Compared to the LVSI-LN- group without metastasis, the top 20 genes showing differential mRNA expression in EEA with LVSI were as follows: BIRC5, COMP, CENPF, AXIN1, CCL3/L1, GMIP, CDC20, CCNE1, CCNB1, EZH2, IL21R, MELK, BLM, FANCA, H2AFX, CCL18, BRCA2, VEGFB, BRCA1, and RRM2 (**Table 3**). For EEA with LN metastasis compared to the LVSI-LN- group, the top 20 differentially expressed mRNAs were: COMP, AXIN1, APH1B, PSMB5, EIF2AK2, ATF3, SLC11A1, MELK, EZH2, BIRC5, CCNE1, ANLN, CENPF, IL1RN, TGFB2, PRLR, AQP9, CCL18, TRIM21, and FGF13 (**Table 4**).

**Table 3:** Top 20 differentially expressed mRNAs in endometrial adenocarcinoma with lymphovascular space involvement (LVSI) compared to expression in the LVSI-LN- group without metastasis.

Gene name	Log2 fold change	P-value	Gene sets
BIRC5	1.95	0.000298	Apoptosis, Cell Proliferation
COMP	3.86	0.000402	Matrix Remodeling and Metastasis, PI3K-Akt
CENPF	1.97	0.000546	Cell Proliferation
AXIN1	0.687	0.000639	Wnt Signaling
CCL3/L1	1.31	0.00137	Cytokine and Chemokine Signaling
GMIP	0.65	0.00143	–
CDC20	1.33	0.00145	Antigen Presentation, Cell Proliferation
CCNE1	1.67	0.00153	Angiogenesis, Cell Proliferation, Metabolic Stress, PI3K-Akt
CCNB1	1.21	0.00158	Cell Proliferation
EZH2	1.05	0.00165	Angiogenesis, Epigenetic Regulation, Metabolic Stress
IL21R	2	0.00205	Cytokine and Chemokine Signaling, JAK-STAT Signaling
MELK	1.25	0.00207	Cell Proliferation
BLM	1.14	0.00222	Apoptosis, Cell Proliferation, DNA Damage Repair
FANCA	1.49	0.00268	DNA Damage Repair
H2AFX	1.14	0.00292	Cell Proliferation, DNA Damage Repair, Epigenetic Regulation, Metabolic Stress
CCL18	1.83	0.00295	Cytokine and Chemokine Signaling
BRCA2	1.23	0.00323	Cell Proliferation, DNA Damage Repair
VEGFB	0.492	0.00352	Angiogenesis, MAPK, PI3K-Akt
BRCA1	0.833	0.00356	Cell Proliferation, DNA Damage Repair, PI3K-Akt
RRM2	0.957	0.00376	Cell Proliferation

LVSI, lymphovascular space involvement; LN, lymph node.

**Table 4:** Top 20 differentially expressed mRNAs in endometrial adenocarcinoma with lymph node (LN) metastasis compared to expression in the lymphovascular space involvement (LVSI-LN-) group without metastasis.

Gene name	Log2 fold change	P-value	Gene sets
COMP	4.46	1.4E-05	Matrix Remodeling and Metastasis, PI3K-Akt
AXIN1	0.849	1.62E-05	Wnt Signaling
APH1B	-1.23	4.54E-05	Notch Signaling
PSMB5	0.718	0.000213	Antigen Presentation, Apoptosis, Cell Proliferation, Costimulatory Signaling, Hedgehog Signaling, NF-κB Signaling
EIF2AK2	-0.659	0.000496	Interferon Signaling
ATF3	-2.6	0.000498	Antigen Presentation
SLC11A1	1.95	0.000669	Myeloid Compartment
MELK	1.31	0.000681	Cell Proliferation
EZH2	1.13	0.000728	Angiogenesis, Epigenetic Regulation, Metabolic Stress
BIRC5	1.77	0.00073	Apoptosis, Cell Proliferation
CCNE1	2.38	0.000805	Angiogenesis, Cell Proliferation, Metabolic Stress, PI3K-Akt
ANLN	1.19	0.000912	Cell Proliferation
CENPF	1.69	0.000913	Cell Proliferation
IL1RN	2.14	0.00102	Cytokine and Chemokine Signaling, Myeloid Compartment
TGFB2	-1.42	0.00103	MAPK, Matrix Remodeling and Metastasis, TGF-β Signaling
PRLR	-1.96	0.00107	Cytotoxicity, JAK-STAT Signaling, PI3K-Akt
AQP9	1.83	0.00109	Metabolic Stress
CCL18	2.06	0.00119	Cytokine and Chemokine Signaling
TRIM21	-0.564	0.00121	Antigen Presentation, Interferon Signaling
FGF13	-1.36	0.00129	MAPK, PI3K-Akt

LVSI, lymphovascular space involvement; LN, lymph node.

Finally, the top 20 differentially expressed mRNAs in the EEAs with LN group compared with EEAs with LVSI were as follows: ATF3, ARNT2, WNT4, HLA-DOA, DUSP1, CD69, TAP1, IL6, STAT1, TGFB2, GBP4, GBP1, MX1, HERC6, PSMB9, NLRC5, EGR1, TAP2, SAMSN1, and DTX3L (**Table 5**).

**Table 5:** Top 20 differentially expressed mRNAs in endometrial adenocarcinoma with lymph node (LN) metastasis compared to those in endometrial adenocarcinoma with lymphovascular space involvement (LVSI).

Gene name	Log2 fold change	P-value	Gene sets
ATF3	-3.02	0.000553	Antigen Presentation
ARNT2	-0.597	0.00147	_
WNT4	1.56	0.0019	Hedgehog Signaling, Wnt Signaling
HLA-DOA	-1.15	0.00208	Antigen Presentation, Immune Cell Adhesion and Migration
DUSP1	-2.1	0.00244	MAPK
CD69	-1.52	0.00285	Costimulatory Signaling
TAP1	-1.4	0.0034	Antigen Presentation
IL6	-2.43	0.00377	Cytokine and Chemokine Signaling, Hypoxia, JAK-STAT Signaling, Metabolic Stress, PI3K-Akt
STAT1	-0.954	0.00407	Cytokine and Chemokine Signaling, Cytotoxicity, Interferon Signaling, JAK-STAT Signaling, Lymphoid Compartment
TGFB2	-0.99	0.00432	MAPK, Matrix Remodeling and Metastasis, TGF-β Signaling
GBP4	-1.59	0.00436	Interferon Signaling
GBP1	-1.51	0.00455	Interferon Signaling
MX1	-1.23	0.00506	Cytotoxicity, Interferon Signaling, Lymphoid Compartment
HERC6	-0.99	0.00573	_
PSMB9	-1.21	0.0059	Antigen Presentation, Apoptosis, Cell Proliferation, Costimulatory Signaling, Hedgehog Signaling, NF-κB Signaling
NLRC5	-0.848	0.00619	_
EGR1	-1.79	0.00678	Costimulatory Signaling, Interferon Signaling, Lymphoid Compartment
TAP2	-0.737	0.00745	Antigen Presentation
SAMSN1	-0.965	0.00866	_
DTX3L	-0.406	0.0087	Antigen Presentation, Notch Signaling

LVSI, lymphovascular space involvement; LN, lymph node.

**Figure 4:**
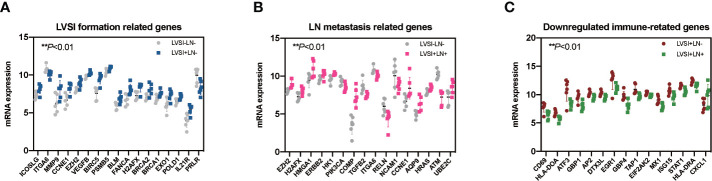
Regulated genes in endometrioid endometrial adenocarcinomas (EEAs) with LVSI and LN metastasis. LVSI, lymphovascular space involvement; LN, lymph node. Points represent the number of cases. Each group has 6 cases. **(A)** Seventeen genes with differential expression in LVSI formation (**P < 0.01). EEAh point represents a case number. Gray dots represent the LVSI-LN- group; dark blue dots represent the LVSI+LN- group. **(B)** Sixteen genes with differential expression in LN metastasis compared to the LVSI-LN- group (**P < 0.01). EEAh point represents a case number. Gray dots represent the LVSI-LN- group; pink dots represent the LVSI+LN+ group. **(C)** Fifteen genes with differential expression in LN metastasis compared to the LVSI+LN- group (**P < 0.01). EEAh point represents a case number. Green dots represent the LVSI-LN- group; red dots represent the LVSI+LN+ group.

Compared with the LVSI-LN- group without metastasis, various regulated signaling pathway-related genes were observed in EEA with LVSI, including those related to the Hedgehog signaling pathways (PSMB5), angiogenesis (CCNE1, EZH2, VEGFB, and MMP9), immune cell adhesion (ICOSLG, ITGA6, and MMP9), cell proliferation and apoptosis (BIRC5, BLM, PSMB5), DNA damage repair (BLM, FANCA, H2AFX, BRCA2, BRCA1, EXO1, POLD1), and JAK-STAT signaling pathways (IL21R, PRLR) (P<0.01) (**Figure 4A**).

Signaling pathway-related genes identified in EEA with LN metastasis compared to the results of the LVSI-LN- group without metastasis were as follows: genes relating to hypoxia (ERBB2, HK1, PIK3CA), metabolic stress (EZH2, CCNE1, AQP9, ERBB2, HK1, HRAS, H2AFX, HMGA1, ATM, PIK3CA, UBE2C), epigenetic regulation (EZH2, H2AFX, HMGA1), and matrix remodeling and metastasis (COMP, TGFB2, ITGA6, RELN, and NCAM1) (P<0.01) (**Figure 4B**).

Immune-related genes in EEA with LN metastasis compared to those in EEA with LVSI were as follows: genes relating to antigen presentation (ATF3, HLA-DOA, TAP1, DTX3L, CXCL1, HLA-DRA), cytotoxicity (STAT1, MX1, ISG15), lymphoid compartment (STAT1, MX1, EGR1, ISG15), interferon signaling (STAT1, GBP4, GBP1, MX1, EGR1, ISG15, EIF2AK2, HLA-DRA), and costimulatory signaling (CD69, EGR1, HLA-DRA) (P<0.01) (**Figure 4C**).

### Distribution Characteristics of Immunocyte Subsets in EEA During LVSI Formation and LN Metastasis

**Figure 5:**
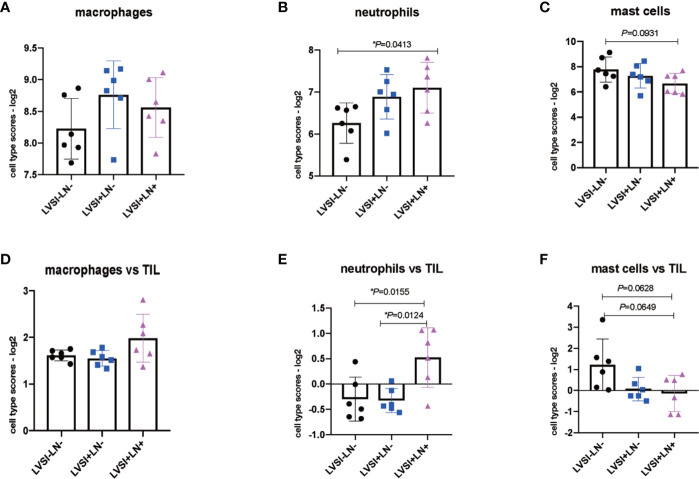
Changes in the distribution of immunocyte subtypes during LVSI formation and LN metastasis of endometrial adenocarcinoma. LVSI, lymphovascular space involvement; LN, lymph node. Points represent the number of cases. Each group has 6 cases. Black, blue, and pink represent LVSI-LN-, LVSI+LN-, and LVSI+LN+, respectively. TIL: tumor infiltrating lymphocyte. Different scores of **(A)** total macrophages, **(B)** total neutrophils, and **(C)** total mast cells are shown for the three groups (LVSI-LN-, LVSI+LN-, LVSI+LN+). The ratios of **(D)** macrophages, **(E)** neutrophils, and **(F)** mast cells in TILs are shown for the three groups (LVSI-LN-, LVSI+LN-, LVSI+LN+). *P < 0.05.

The results revealed that immunocyte subtypes and genes relating to immune surveillance and immune escape, such as NK cells, CD8 T cells, Treg cells, DCs, exhausted CD8, TIL count, PD-L1, and CTLA4, showed no significant differences in expression in the absence of LVSI or with LVSI and LN metastasis in EEA progression (**Supplementary Figures 2** and **3**). However, total neutrophils were upregulated in the LVSI+LN+ group compared to the LVSI-LN- group (P<0.05) (**Figure 5B**). Total macrophages also showed an upward trend, though not statistically significant. Whereas mast cells showed a downward trend, although there was no statistical significance (P=0.093) (**Figures 5A, C**). The ratio of neutrophils in TIL was up-regulated in LVSI+LN+ group, compared to LVSI+LN- and LVSI-LN- group (**Figures 5E, F**). The ratio of macrophages in TIL showed an upward trend in LVSI+LN+ group, compared to LVSI+LN- group and LVSI-LN- group (**Figure 5D**).

**Figure 6:**
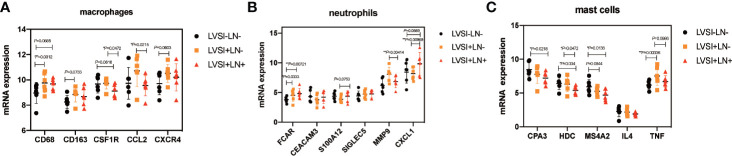
Changes in the distribution of immunocyte subtype-related genes during LVSI formation and LN metastasis of endometrial adenocarcinoma. LVSI, lymphovascular space involvement; LN, lymph node. Points represent the number of cases. Each group has 6 cases. Black, orange, and red represent LVSI-LN-, LVSI+LN-, and LVSI+LN+, respectively. The mRNA expression of **(A)** 5 macrophage-related genes, **(B)** 6 neutrophil-related genes, and **(C)** 5 mast cell-related genes is shown in the three groups (LVSI-LN-, LVSI+LN-, LVSI+LN+). *P < 0.05, ** *P* < 0.01.

We further analyzed gene expression relating to macrophages, neutrophils, and mast cells. Macrophage-related gene CD68 was upregulated in the LVSI+LN group compared to the LVSI-LN- group. However, CSFIR and CCL2 were downregulated in the LVSI+LN+ group compared to the LVSI+LN- group (**Figure 6A**). Neutrophil-related gene FCAR was upregulated in the LVSI+LN- group and LVSI+LN+ group compared to the LVSI-LN- group. Neutrophil-related gene CXCL1 were upregulated in the LVSI+LN+ group compared to those in the LVSI+LN- group (**Figure 6B**). Among mast cell-related genes, tumor necrosis factor (TNF) was upregulated in the LVSI+LN- group compared to the LVSI-LN- group. Moreover, the mast cell-related genes CAP3, HDC, and MS4A2 were downregulated in the LVSI+LN+ group compared to those in the LVSI-LN- group (**Figure 6C**).

### HE Staining and Immunohistochemical Staining Confirmed the Expression of Hub Genes and Immunocytes

**Figure 7:**
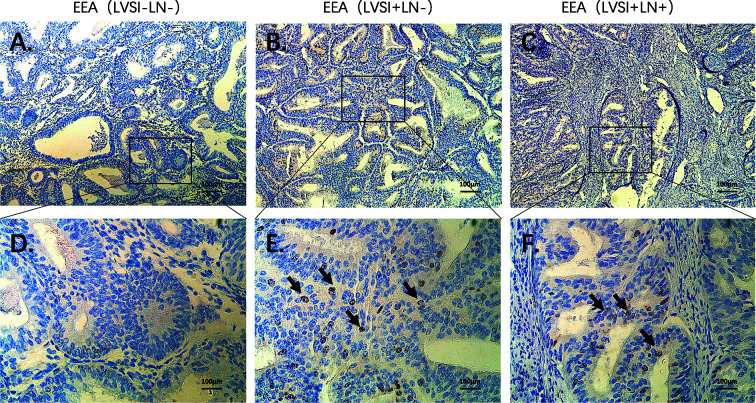
Immunohistochemical staining of BIRC5 during LVSI formation and LN metastasis of endometrioid endometrial adenocarcinomas (EEAs). **(A)** LVSI-LN- group. 100X magnification **(B)** LVSI+LN- group. 100X magnification. **(C)** LVSI+LN+ group. 100X magnification. **(D)** LVSI-LN- group. 400X magnification. **(E)** LVSI+LN- group. 400X magnification. **(F)** LVSI+LN+ group. 400X magnification. The black arrow indicates brown black positive expression particles.

**Figure 8:**
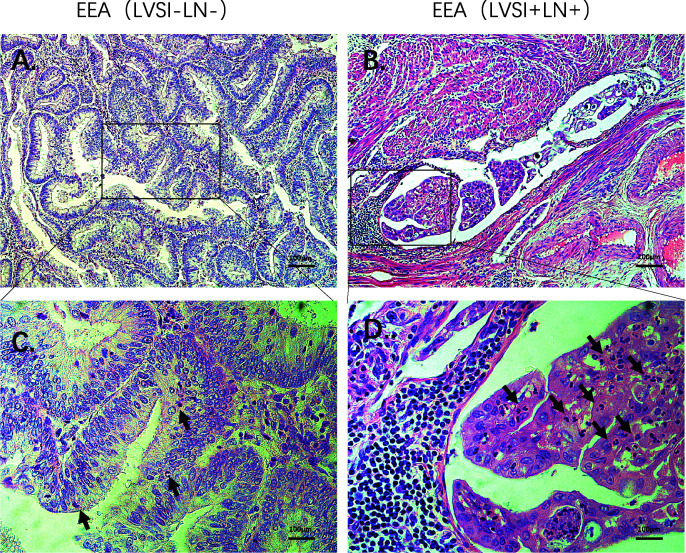
HE staining of neutrophils during metastasis of endometrioid endometrial adenocarcinomas (EEAs). **(A)** LVSI-LN- group. 100X magnification **(B)** LVSI+LN+ group. 100X magnification. **(C)** LVSI-LN-group. 400X magnification. **(D)** LVSI+LN-+group. 400X magnification. The black arrow represents neutrophils.

**Figure 9:**
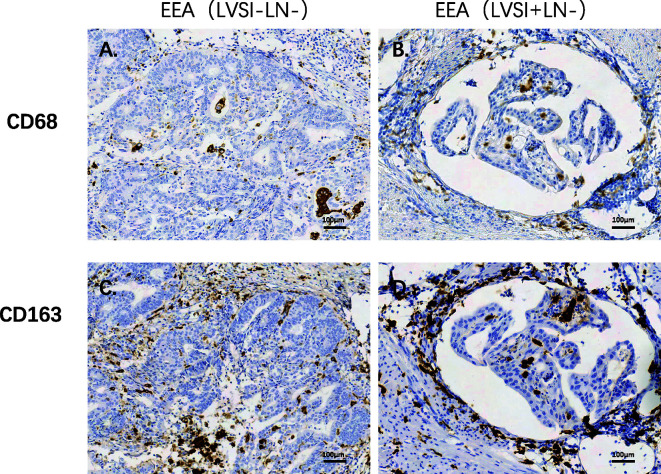
Immunohistochemical staining of macrophages-related genes during LVSI formation of endometrioid endometrial adenocarcinomas (EEAs). **(A)** CD68 protein expression in LVSI-LN- group. 200X magnification **(B)** CD68 protein expression in LVSI+LN- group. 200X magnification **(C)** CD163 protein expression in LVSI-LN- group. 200X magnification. **(D)** CD163 protein expression in LVSI+LN- group. 200X magnification.

Compared to the LVSI-LN- group without metastasis, BIRC5 related to cell proliferation was in top 20 gene in LVSI+LN- group and LVSI+LN+ group. So, we further verified the BIRC5 *in situ* protein expression in EEAs tissues. We observed that the brown black positive granules were expressed in the LVSI+LN- group and LVSI+LN+ group, but were absent in the LVSI-LN- group tissues without LVSI and LN metastasis (**Figure 7**). The total number of neutrophils was significantly increased in the LVSI+ LN+ group compared with the LVSI-LN- group observed in the HE staining EEAs sections (**Figure 8**). The expression of macrophage-associated genes CD68 and CD163 was stronger and more positive in LVSI+LN- group, compared to LVSI-LN- group (**Figure 9**). The expression trends of BIRC5, CD68, CD163, and neutrophiles in tissues detected by immunohistochemistry and HE staining were consistent with the result of nanostring mRNA analysis.

## Discussion

T cell-mediated adaptive immunity plays an active role in the anti-tumor process. DCs, macrophages, and B cells present tumor antigens to CD4+ helper T cells. In turn, they cooperate to induce CD8+ T cells and B cells as well as activate NK cells and macrophages. These pathways ultimately eliminate tumor cells through the CD8+ cytotoxic T cell-dependent apoptosis pathway. Moreover, an imbalance in T cell-mediated responses plays a negative role in cancer progression, leading to an immunosuppressive environment and tumor escape (15–17). High TIL levels occur in anti-tumor immune response and indicate a good prognosis in gastric cancer and breast cancer (18, 19). Moreover, regulation of lymph node function by NK cells is associated with prognosis in patients with stage II colon cancer (20). Study have shown that the average fluorescence intensity of CD8 staining in peripheral blood of patients with grade 3 EEC is lower than that of healthy donors. Cytotoxic T cells with decreased CD8 expression were positively correlated with EEAs (21). Endometrial tumor microenvironment reduces the recruitment of NK cells and changes the phenotype and function of NK cells (22). The profiles of immune infiltration NK cells, DCs and CD8+T cells showed associated with patients survival in TCGA uterine cancer cohort (23). This study showed that immunocytes (CD8, NK cells and DC) tended to recede in the advanced stage of LN metastasis compared to those in the LVSI+LN- group, leading to immunotolerance or immunosuppression. However, due to the limited sample size, these immune cells did not show significant difference. The obtained expression profile of immune microenvironment regulation during LN metastasis in endometrial carcinoma was consistent with the results of previous studies.

In this study, the ratio of macrophages in TILs showed an upward trend in the LVSI+LN+ group compared to that in the LVSI+LN- and LVSI-LN- groups. Various studies have shown that tumor-associated macrophages (TAMs) in endometrial carcinoma are associated with increases in LVSI and LN metastasis and a poor survival outcome (24). TAMs are polarized M2 macrophages, which secrete cytokines, chemokines, and growth factors to promote EC development and inhibit the anti-tumor immune system. CTHRC1 increases the recruitment of M2-like macrophages, prompting myometrial invasion in endometrial carcinoma by regulating the integrin-Akt signaling pathway (25). TAM reduced the ERα expression in EC cells *via* HOXB13 by increasing CXCL8 secretion, thus promoting EC invasion and metastasis (26). Invasive macrophages induce ERα expression through epigenetic mechanism mediated by IL17A, which makes endometrial cancer cells sensitive to estrogen (27).

For a long time, the function of neutrophils in the tumor microenvironment has been ignored. Recently, accumulating studies have shown that neutrophils are involved in promoting tumor progression and metastasis (28, 29). Wculek & Malanchi (30) showed that neutrophils are involved in lung metastasis in mouse breast cancer models, whereas drug- or gene-mediated suppression of the leukotriene-generating enzyme arachidonate 5-lipoxygenase abolished neutrophil-associated pro-metastatic functions. During the formation of the tumor metastasis microenvironment, increased secretion of CXCL1 and CXCL2 by endothelial cells and megakaryocytes promotes the release of neutrophils into circulation *via* the regulation of CXCR2 signaling. Neutrophil-derived MMP9 is more likely to activate and participate in pre-tumor functions (31). Neutrophils produce rEEAtive oxygen species, which cause DNA damage, genomic instability, and gene mutations in precancerous epithelial cells, thus promoting the carcinogenic transformation (32). For the first time, this study found that neutrophils showed a rising trend when LN metastasis develop in EC. Compared to the LVSI-LN- group, VEGFB and MMP9 showed higher expression in the LVSI+LN- group. VEGFB, MMP9, and neutrophils may cooperatively regulate angiogenesis in the primary metastasis of endometrial carcinoma. Moreover, CXCL1 mRNA expression was upregulated in the LVSI+LN+ group compared to that in the LVSI+LN- group, suggesting that the high expression of chemokine CXCL1 may be involved in the recruitment of neutrophils to lymph vessels during the progression of lymph node metastasis in EEA.

Studies have also found that mast cells play a dual role in regulating cancer progression (33, 34). First, mast cells participate in the internal and acquired immune process, promote DC migration and maturation, and interact with T and B cells. Additionally, they can promote the release of inflammatory factors such as TNF-α, MIP, and MCP and participate in the formation of an anti-tumor inflammatory microenvironment. However, tryptases secreted by mast cells can promote angiogenesis and lymphangiogenesis, providing a platform and channel for tumor metastasis (33). Accumulating studies have shown controversial and variable results regarding the role of mast cells in different types of tumors and in different stages of cancer progression. Mast cells derived from adipose tissue promote apoptosis of breast cancer cells by secreting TNF-α and granulocyte-macrophage colony-stimulating factor (35). A previous study revealed that activated mast cells were downregulated in breast cancer tissues of the LN-positive group compared to those of the LN-negative group (36). Conversely, another study found that mast cell density was increased in metastatic LNs in breast cancer (37). In this study, we found downregulated expression of mast cell-related genes in both LVSI formation and LN metastasis, suggesting that mast cells play a negative role in regulating immune response tolerance and LN metastasis in EEA.

The important upregulated genes involved in LVSI were BIRC5, CDC20, CCNE1, EZH2, MMP9, et al. It is clear that cell proliferation, angiogenesis, immune cell adhesion, DNA damage, and JAK-STAT signaling pathways were involved in EC with LVSI formation. Furthermore, the antigen presentation-related genes ATF3, HLA-DOA, TAP1, DTX3L, and HLA-DRA were significantly decreased in the LVSI+LN+ group compared to the LVSI+LN- group. Moreover, genes relating to costimulatory signaling (CD69, EGR1, and HLA-DRA), interferon signaling (STAT1, GBP4, GBP1, MX1, EGR1, ISG15, EIF2AK2, and HLA-DR), and the lymphoid compartment (STAT1, MX1, EGR1, and ISG15) were significantly downregulated in the LVSI+LN+ group compared to the LVSI+LN- group. Overall, the immune surveillance system was devastated during the development of LN metastasis in endometrial carcinoma. However, metabolic stress, matrix remodeling and metastasis, PI3K-AKT, and hypoxia signaling pathways were always activated during LN metastasis.

In conclusion, this study found that ECs with metastatic LNs showed significant immunosuppressive effects. Some oncogenes as well as genes relating to matrix remodeling, hypoxia, macrophages, and neutrophil signatures showed higher expression, suggesting their potential for use as therapeutic targets and offering new immunotherapy strategies in EEA during LN metastasis.

## Data Availability Statement

According to national legislation/guidelines, specifically the Administrative Regulations of the People’s Republic of China on Human Genetic Resources (http://www.gov.cn/zhengce/content/2019-06/10/content_5398829.htm, http://english.www.gov.cn/policies/latest_releases/2019/06/10/content_281476708945462.htm), no additional raw data is available at this time. Data of this project can be accessed after an approval application to the China National Genebank (CNGB, https://db.cngb.org/cnsa/). Please refer to https://db.cngb.org/, or email: CNGBdb@cngb.org for detailed application guidance. The accession code CRA003336 (http://bigd.big.ac.cn/gsa/s/L5wcNuAp) should be included in the application.

## Ethics Statement

The studies involving human participants were reviewed and approved by the ethics committee of Peking University People’s hospital. The patients/participants provided their written informed consent to participate in this study. Written informed consent was obtained from the individual(s) for the publication of any potentially identifiable images or data included in this article.

## Author Contributions

YC chiefly wrote this manuscript and collected clinical data and samples. XZ was primarily responsible for pathological diagnosis. ZW gave advice on the selection of clinical samples. JW was mainly responsible for project design. All authors contributed to the article and approved the submitted version.

## Funding

The National Natural Science Foundation of China (Grant no. 81802607, 81874108, and 81672571), the Beijing Municipal Natural Science Foundation (Grant no.7202213), the Fund for Fostering Young Scholars of Peking University Health Science Center (Grant no. BMU2017PY011), the Research and Development Fund of Peking University People’s Hospital (Grant no. RDY2017-12), and National Key Technology R&D Program of China (Grant No. 2019YFC1005200 and 2019YFC1005201).

## Conflict of Interest

The authors declare that the research was conducted in the absence of any commercial or financial relationships that could be construed as a potential conflict of interest.

## Supplementary Material

Supplementary Figure 1 Some signaling pathways showed no significant changes in the groups without LVSI or with LVSI formation or LN metastasis in endometrial adenocarcinoma progression. LVSI: lymphovascular space involvement; LN: lymph node. Points represent the number of cases. Black, green, and purple represent LVSI-LN-, LVSI+LN-, and LVSI+LN+, respectively. Scores of **(A)** cytokine and chemokine signaling, **(B)** myeloid compartment, **(C)** autophagy, **(D)** TGF-β signaling, **(E)** MAPK, **(F)** NF-κB signaling, **(G)** Notch signaling, and **(H)** Wnt signaling are shown in the three groups (LVSI-LN-, LVSI+LN-, LVSI+LN+).

Supplementary Figure 2 Some immunocyte subtypes showed no significant changes in the groups without LVSI or with LVSI formation or LN metastasis in endometrial adenocarcinoma progression. LVSI: lymphovascular space involvement; LN: lymph node; TIL: tumor-infiltrating lymphocyte. Points represent the number of cases. Black, blue, and pink represent LVSI-LN-, LVSI+LN-, and LVSI+LN+, respectively. Scores for **(A)** CD8, **(B)** natural killer cells, **(C)** dendritic cells, **(D)** Tregs, and **(E)** exhausted CD8 cells are shown in the three groups (LVSI-LN-, LVSI+LN-, LVSI+LN+). Ratios of **(F)** CD8, **(G)** natural killer cells, **(H)** dendritic cells, **(I)** Tregs, and **(J)** exhausted CD8 cells are shown in the three groups (LVSI-LN-, LVSI+LN-, LVSI+LN+).

Supplementary Figure 3 TIL counts and PD-L1 expression showed no significant changes in the groups without LVSI or with LVSI formation or LN metastasis in endometrial adenocarcinoma progression. LVSI: lymphovascular space involvement; LN: lymph node; TIL: tumor-infiltrating lymphocyte. Points represent the number of cases. Black, blue, and pink represent LVSI-LN-, LVSI+LN-, and LVSI+LN+, respectively. **(A)** TIL count scores, **(B)** PD-L1 mRNA expression, and **(C)** CTLA4 mRNA expression are shown in the three groups (LVSI-LN-, LVSI+LN-, LVSI+LN+).
