# Supplementary figures and images for: Reconstruction of Immune Microenvironment and Signaling Pathways in Endometrioid Endometrial Adenocarcinoma During Formation of Lymphovascular Space Involvement and Lymph Node Metastasis
Source: Front Oncol. 2020 Dec 9;10:595082. doi: 10.3389/fonc.2020.595082 (PMC7756104; doi:10.3389/fonc.2020.595082)

Suppl. figure 1.

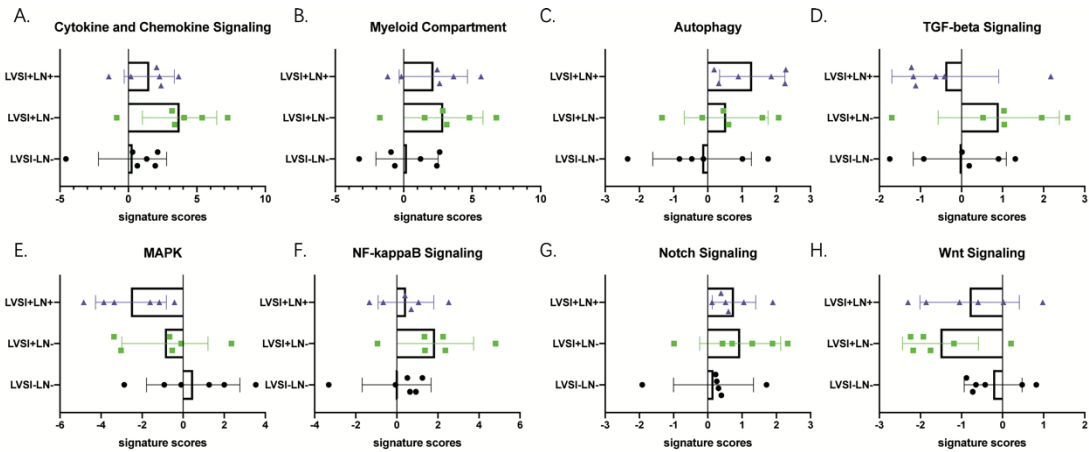

Suppl. figure 2.

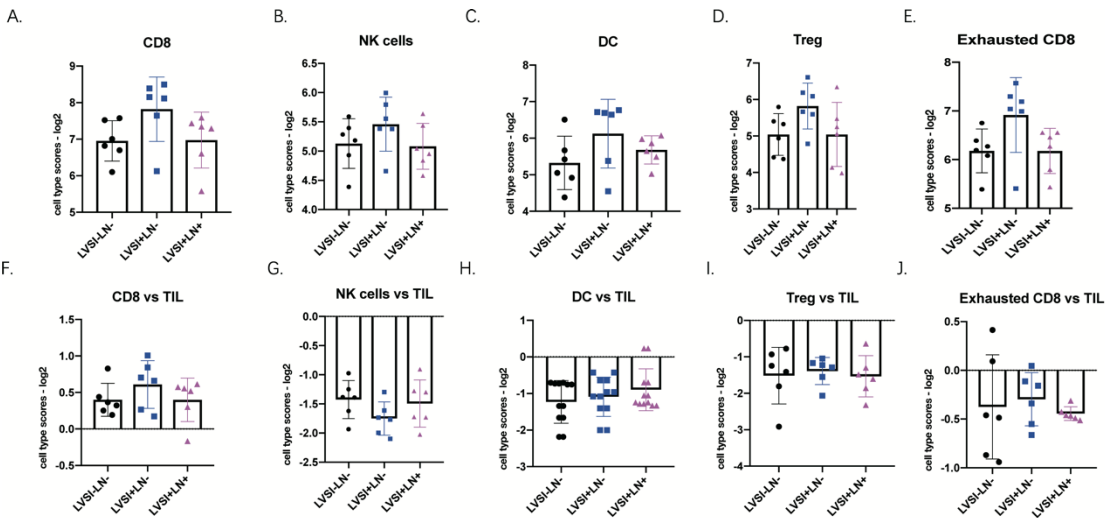

Suppl. figure 3.

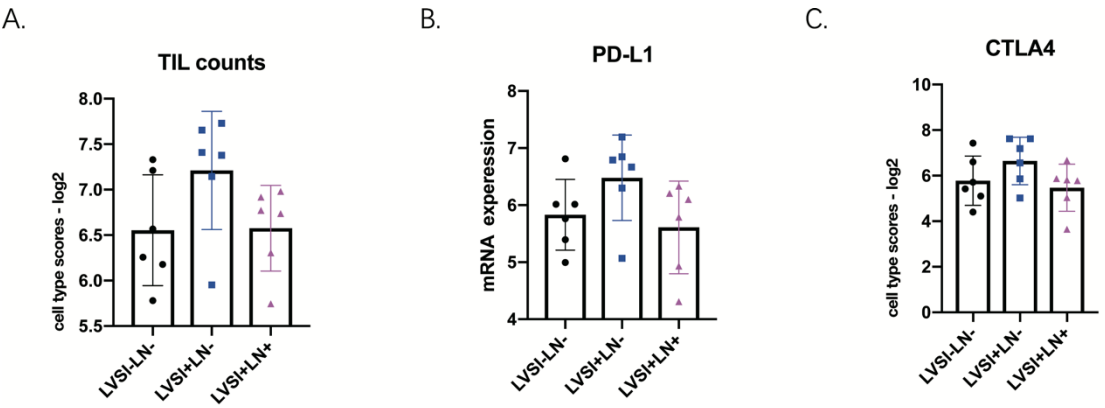

Supplement: Supplementary Figure 1 — Some signaling pathways showed no significant changes in the groups without LVSI or with LVSI formation or LN metastasis in endometrial adenocarcinoma progression. LVSI: lymphovascular space involvement; LN: lymph node. Points represent the number of cases. Black, green, and purple represent LVSI-LN-, LVSI+LN-, and LVSI+LN+, respectively. Scores of (A) cytokine and chemokine signaling, (B) myeloid compartment, (C) autophagy, (D) TGF-β signaling, (E) MAPK, (F) NF-κB signaling, (G) Notch signaling, and (H) Wnt signaling are shown in the three groups (LVSI-LN-, LVSI+LN-, LVSI+LN+). [file DataSheet_1.pdf]
